# Promoting endoscopists' health through cutting‐edge motion analysis technology: Accuracy and precision of ergonomic motion tracking system for endoscopy suite (EMTES)

**DOI:** 10.1002/1348-9585.12355

**Published:** 2022-09-07

**Authors:** Hiroaki Ono, Yasuki Hori, Mafu Tsunemi, Ippei Matsuzaki, Kazuki Hayashi, Michihiro Kamijima, Takeshi Ebara

**Affiliations:** ^1^ Department of Occupational and Environmental Health Nagoya City University Graduate School of Medical Sciences/Medical School Nagoya Japan; ^2^ Department of Gastroenterology and Metabolism Nagoya City University Graduate School of Medical Sciences Nagoya Japan; ^3^ Department of Nursing Yamashita Hospital Ichinomiya Japan; ^4^ Department of Gastroenterology Yamashita Hospital Ichinomiya Japan

**Keywords:** accuracy, endoscopist, motion tracking, musculoskeletal disorders, occlusion, precision

## Abstract

**Objectives:**

Endoscopists often suffer from musculoskeletal disorders due to posture‐specific workloads imposed by precise maneuvering or long procedural duration. An ergonomic motion tracking system for endoscopy suite (EMTES) was developed using Azure Kinect sensors to evaluate the occlusion, accuracy, and precision, focusing mainly on upper and lower limb movements.

**Methods:**

Three healthy male participants pointed the prescribed points for 5 s on the designated work envelopes and their coordinates were measured. The mean occlusion rate (%) of the 32 motion tracking landmarks, standard deviation (SD) of distance and orientation, and partial regression coefficient (β) and *R* ^2^ model fit for accuracy were calculated using the time series of coordinates data of the upper/lower limb movements.

**Results:**

The mean occlusion rate was 5.2 ± 10.6% and 1.6 ± 1.4% for upper and lower limb movements, respectively. Of the 32 landmarks, 28 (87.5%) had occlusion rates of 10% or less. The mean SDs of 4.2 mm for distance and 1.2° for orientation were found. Most of the *R* ^2^ values were over 0.9. In the case of right upper/lower limb measurement for orientation, β coefficients ranged from 0.82 to 1.36.

**Conclusion:**

EMTES is reliable in calculating occlusion, precision, and accuracy for practical motion‐tracking measurements in endoscopists.

## 1 INTRODUCTION

Endoscopists perform specific maneuvers and face constraints due to their posture, such as adjusting the tip of angulation controls of the endoscope using their left thumb and exerting a strong torque using their right wrist for operating the endoscope. Such frequent, repetitive maneuvers and postural habits may lead to musculoskeletal disorders (MSDs) in endoscopists. In fact, recent reports indicate that the incidence of MSDs among endoscopists has reached 52.9–79.1%, with some disorders and has resulted in a temporary leave of absence.^1^, ^2^, ^3^

Recent research^4^ has attempted to reveal the factors causing endoscopic upper limb MSDs using a biomechanical approach focusing on thumb pinch force and forearm muscle loads of the extensor carpi radialis and flexor digitorum superficialis muscles. MSDs encompass‐related symptoms such as work‐related upper limb disorder, repetitive strain injury, and occupational overuse syndrome that develop as a result of repetitive movements, awkward/constrained postures, and the impact of external forces with operating tools. This implies the need to grasp such movements and postures of endoscopists objectively in a real setting, as well as their muscle force/loads.

Therefore, we developed an ergonomic motion tracking system for the endoscopy suite (EMTES, a beta version currently under development) using Azure Kinect sensors (Microsoft, Redmond, VA), a real‐time human motion tracking technology easy to implement with low cost, intended for use with a real endoscopy suite setting. The EMTES is intended for grasping movements and postures of endoscopists relevant to work‐related upper limb disorder and repetitive strain injury, as well as neck, low back, and shoulder MSDs. However, occlusion, i.e., an inability to accurately track human motion due to blind spots between the subject and sensors, is dependent on the actual situation. Furthermore, measurement accuracy and precision need to be verified as well, as these might be affected by the use of a real endoscopy suite in the presence of various medical devices and workstation layouts.

The purpose of this study was to verify the occlusion rates of the EMTES when measuring in a real endoscopy suite setting and to evaluate the accuracy and precision, mainly focusing on upper and lower limb movements.

## 2 METHODS

### 2.1 Participants

Three healthy male participants (mean age 47.3 ± 4.7, height 170.7 ± 3.5 cm, body mass index 25.8 ± 5.8 kg/m^2^) who have no history of upper/lower limb musculoskeletal disorders voluntarily participated in this study. The study was approved by the Institutional Review Board of Nagoya City University (approval no. 46–20–0003).

### 2.2 Experimental apparatus

**FIGURE 1:**
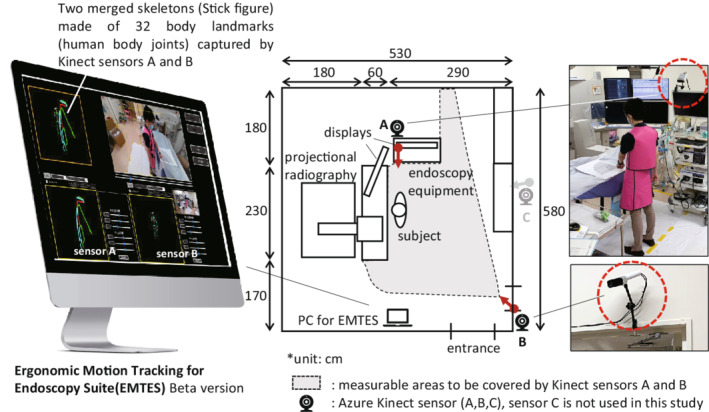
Ergonomic Motion Tracking for Endoscopy Suite (EMTES).

The EMTES was set up to measure the 3‐dimensional coordinates of 32 body landmarks of up to four participants simultaneously with a maximum of three sensors placed on the right, rear, and left rear sides of the participant (A, B, and C, sampling rate: 15 frames per second [fps], details in Figure 1). Only two of the three sensors (A and B) were activated in this study in order to reduce the data processing loads and to increase measurement stability. Sensor A, which was mainly intended for tracking upper limb movements, was fixed at a height of 195 cm on the display using an arm tripod, with the distance of the participant set within 65 cm from the right lateral side. Sensor B, which was mainly intended for tracking the whole body and lower limb movements, was fixed at a height of 185 cm from the floor, with the distance to the participant to be within 400 cm from the left posterior side. Both positions of the camera were considered based on the characteristics of the operation when operating the endoscope.

### 2.3 Procedures

For verifying the extent to which landmarks were not visible when taking basic movements of the upper/lower limbs of a participant, reference data needs to be established. Moreover, of the 32 markers used, fingertip (HandTip, defined as the landmark name) and toe (Foot) play an important role in grasping endoscopy‐specific movements when using the left thumb and right wrist for operating the endoscope and to monitor sustained standing postures under restricted working areas. Hence, to reveal the occlusion rate, precision, and accuracy of the horizontal reach/work envelopes, we prepared a sheet of concentric semi‐circles with points at 0, 15, and 30 cm in five orientations of 0°, 30°, 45°, 60°, and 90° for upper limb movements. A similar sheet but with points at 10, 20, and 30 cm was constructed for the lower limb movements.

The sheet for the upper limb measurement was placed on the endoscopy examination table, and the participant stood in front of it and sequentially pointed to each prescribed point (for 5 s/point) on the sheet with his right fingertip, followed by the left one. Likewise, a sheet for the lower limb movement was placed on the floor, and the participant stood in front of it and sequentially pointed to each prescribed point on the sheet with his right/left toes. Assuming an endoscopic retrograde cholangiopancreatography (ERCP) procedure, participants were requested to wear radiation protective devices such as a lead apron and glasses.

### 2.4 Data analysis

The time‐series coordinate data (15 Hz) from the two sensors were recorded and merged in the EMTES with three levels of confidence: 0 (low), 1 (medium), and 2 (high) provided/defined by the Azure Kinect software development kit algorism. Of such level data, we extracted only data with level 2 as valid data for verifying the occlusion rates of each landmark. Mean occlusion rate (%) was calculated by each individual, defined as the extent to which each landmark is missing during the measurement time period, and finally, the mean ± SD for each landmark was obtained from the three participants.

Two types of measurement errors, precision, and accuracy, were adapted. Precision reflects the degree of reproducibility of the measurement, i.e., the within‐measurement variation. On the other hand, accuracy indicates the extent of the closeness of the measured value to a true value. As for the precision of measurements, since the time‐series of coordinate data obtained from the sensors have tracking noises, the so‐called “jitter”,^5^ we used standard deviations (SDs) of distance and orientation when measuring upper/lower movements as a metric of such variations in terms of jitter. Note that the orientation error is calculated based on the angle between the horizontal line of 0° and each point which is generated from at least three points, including the center point of a semicircle. SD distribution was classified into quartiles. The accuracy indicating the systematic errors in distance and orientation between the sensor‐measured and actual values (defined in the sheet of concentric semi‐circles) were evaluated by the partial regression coefficient (β) and *R* ^2^ model fit.

## 3 RESULTS

**FIGURE 2:**
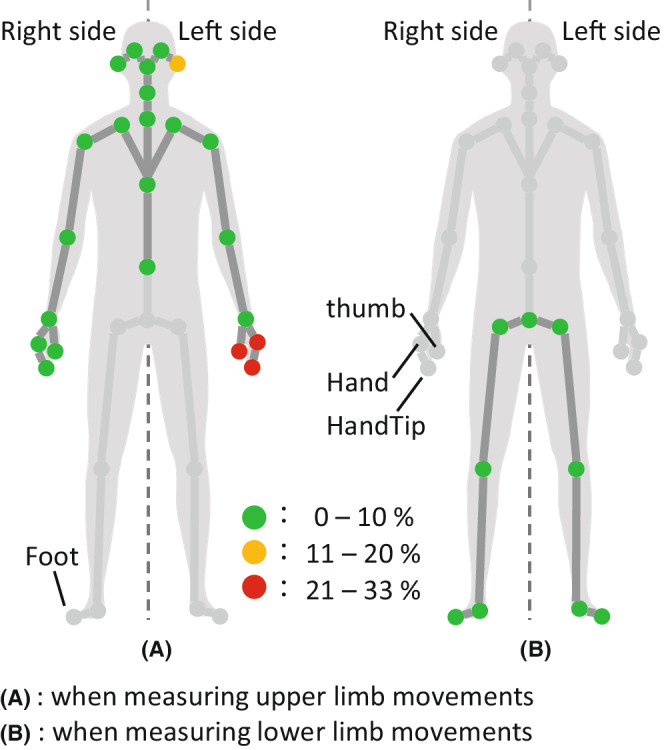
Occlusion rates of 32 body landmarks (*n* = 3, frontal plane, anterior part).

The mean occlusion rate was 5.2 ± 10.6% and 1.6 ± 1.4% for upper and lower limb movement, respectively. Of the 32 landmarks, 28 (87.5%) had occlusion rates of 10% or less, indicating low occlusion rates (Figure 2). On the other hand, the occlusion rates for the three hand‐related landmarks, left‐hand, left‐hand tip, and left thumb were relatively high: 30.3%, 32.1%, and 30.7%, respectively.

**FIGURE 3:**
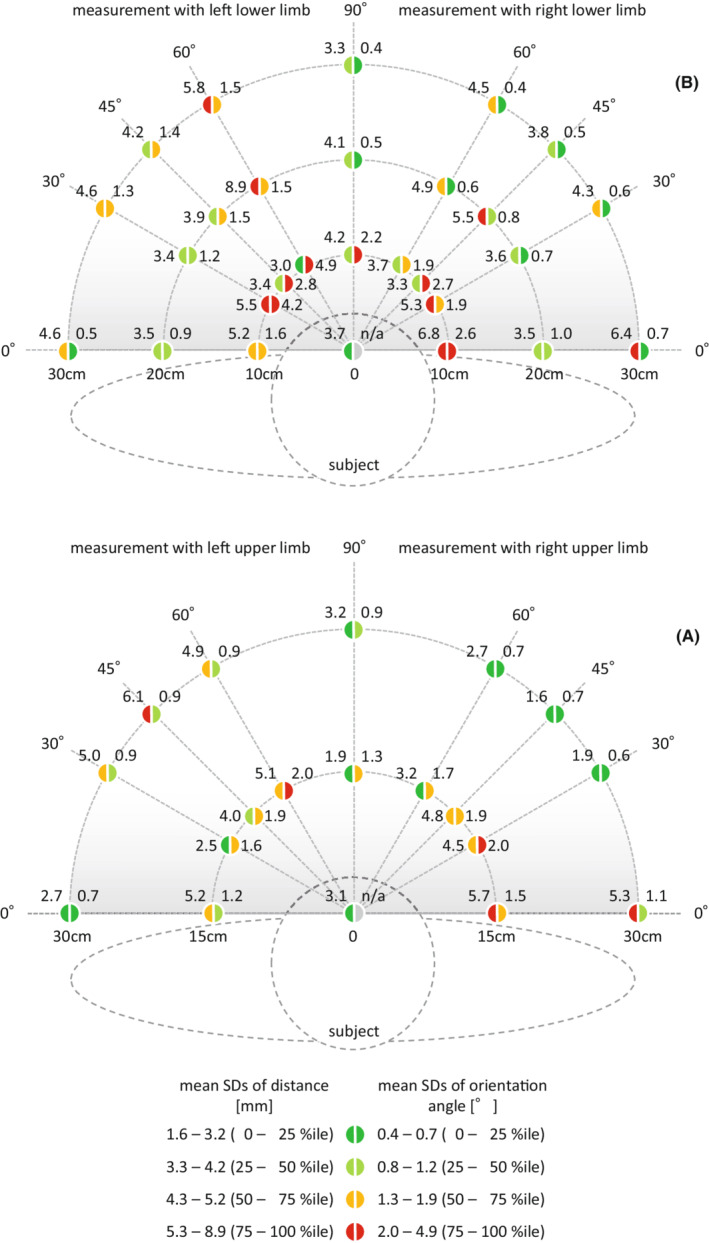
Measurement precision on the horizontal reach/work envelope: mean SDs of distance and orientation when measuring with right−/left upper/lower limbs (*n* = 3).

The mean SD of distance was 4.2 mm (minimum: 1.6 mm, maximum: 8.9 mm) and the mean SD of angles was 1.2° (minimum: 0.4°, maximum: 4.9°). The precision of each point on the horizontal reach/work envelope when measuring upper/lower limb movements is illustrated as infographics (Figure 3). The relatively high fluctuation of values (<75 percentile, points shaped as red semicircles) tended to be near the front and ahead of the left side of the participants which was common in both the upper and lower limb measurements.

**FIGURE 4:**
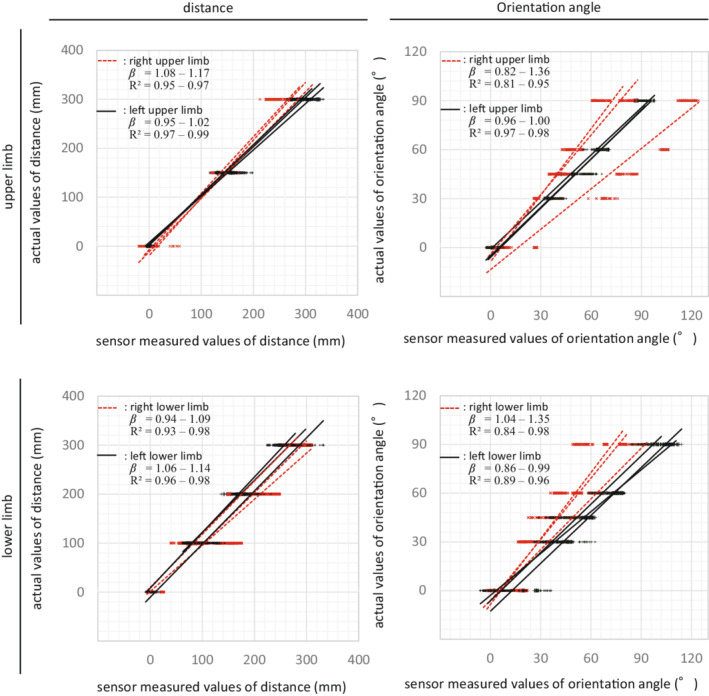
Measurement accuracy between sensor‐measured and actual values on the distance and orientation angle under the conditions of upper/lower limb movements.

Single regression analyses of accuracy metrics showed that all results reached a *R* ^2^ over 0.9, except in the case of the right upper/lower limb angular (*R* ^2^ > 0.81) movement (Figure 4). β coefficients were around 1 (0.94–1.17) for the distance measurements. In the case of the orientation angles, β coefficients ranged from 0.82 to 1.36, indicating that the measured angles had a systematic tendency to be over‐/under‐estimated compared to their actual values.

## 4 DISCUSSION

The two activated sensors provided sufficiently low occlusion rates of less than 10% for 87.5% of the landmarks. Seo et al.^6^ proposed the placement of a Kinect sensor for measuring the upper limb range of motion accurately to be elevated 45° in front and tilted toward the participant. However, another study^7^ suggests that placing a Kinect sensor right in front of a participant is not a good choice for complex upper limb tasks. Placing a sensor at the contralateral side of a participant with an orientation around 30°–45° is recommended for minimizing occlusion in upper limb movements in case of a single‐sensor measurement.^7^, ^8^ A real endoscopy suite setting has restrictions when placing sensors. Additionally, in the case of the EMTES, which merges multiple sensor data, the location of the sensors needs to be fixed to determine spatial mapping based on the reference coordinate (0, 0, 0) between different sensors. We first tried to have a set sensor A on the main display located toward the right and front of the participants; however, the display is flexibly adjusted during the ERCP procedure. Hence, we had no choice but to place sensor A at the right side of the participant. Such condition yields the points close to the trunk and the left side of the body (contralateral side from sensor A) and tends to be lost by self‐occlusion. This was mainly why the three hand‐related landmarks had relatively high occlusion rates compared to the others. Thus, the combination of placements of multiple sensors under a real setting should be further researched to minimize occlusion.

As for the precision, some relatively high fluctuating values were found near the front and ahead of the left side of the participants (Figure 3); nevertheless, the mean SDs were up to 8.9 mm for distance and 4.9° for orientation. A previous study^9^ shows that the SDs of distance measurement by the Kinect sensor ranged from 4.8 to 5.9 mm. Angular measurements also had systematic biases ranging from 14° ± 4° to 36° ± 5°, depending on the measurement situation.^6^ These experiments are not exactly similar to ours though, yet our results showed a similar level of precision. The precision level is enough to be applied and feasible for measuring endoscopists' movement in a real endoscopy setting. For example, in the case of a marker‐based motion capture system (such as VICON), the participant needs to put markers on the body surface and be measured using 8–16 large optical sensors arranged in a circular pattern in a dedicated studio. Even though this system has an extremely high‐level precision, reaching SDs of 0.5 mm/0.34°,^10^ it is quite expensive and is not an available option to perform the measurements outside of a laboratory. Another possible solution might be OpenPose, which uses only video images and does not need to use depth sensor data. It has high applicability and is easy to use but has an absolute error of 30 mm or less.^11^ Compared to these, EMTES can be considered a good available choice in terms of cost‐effectiveness.

As for the accuracy, most of the sensor‐measured distance/orientation data reflected the actual values, reaching a *R* ^2^ of more than 0.9. However, in the case of orientation angle measurements, a systematic error was found to be over‐/under‐estimated, indicating that careful consideration is needed when analyzing data. Such systematic error needs to be adjusted using the regression formula by obtaining reference data prior to each measurement.

### 4.1 Limitations of this study

The EMTES under development achieves stable measurement when using only two sensors so far. If three cameras were available, the occlusion could have been further reduced. Although only one participant for one experiment was tested in this study, the occlusion rate might be even higher due to the presence of endoscopy nurses or medical staff simultaneously in an actual procedural setting. The actual layout of the endoscopy room is different depending on each institution. Further research on the accuracy and precision of EMTES under various endoscopy suite conditions using multiple cameras needs to be accumulated. Especially, it should be that the camera's position is considered based on the characteristics of the endoscopic operation when the camera setting. The type of task assessed in this involves is near‐static; therefore, further testing for dynamic movements is warranted.

### 4.2 Conclusion

We revealed that the EMTES performs sufficiently well in terms of occlusion, precision, and accuracy for practical motion‐tracking measurements of endoscopists in an endoscopy workstation. We aim to make the EMTES, which has cutting‐edge sensing technologies in addition to the advantage of its low‐cost and non‐invasiveness to be widely available to the public in near future and to make it available in each medical institution to ensure endoscopists' health.

## DISCLOSURE

*Approval of the research protocol*: The study was approved by the Institutional Review Board of Nagoya City University (approval no. 46–20–0003).*Informed consent*: All participants were informed about the purpose and content of the study and gave their informed consent to participate in the study.*Registry and the Registration*: N/A*Animal Studies*: N/A. *Conflict of Interest*: The authors declare no conflicts of interest associated with this manuscript.

## AUTHOR CONTRIBUTION

Conception and design; Hiroaki Ono, Yasuki Hori, and Takeshi Ebara, Analysis and interpretation of the data; Hiroaki Ono, Yasuki Hori, Mafu Tsunemi, Ippei Matsuzaki, Kazuki Hayashi, and Takeshi Ebara, Drafting of the article; Hiroaki Ono and Takeshi Ebara, Critical revision of the article for important intellectual content; Michihiro Kamijima and Takeshi Ebara. All authors approved the final draft of the manuscript.

## FUNDING INFORMATION

The study was supported by the Nitto Foundation (grant no. JOSE202100) and the Japan Science and Technology Agency (JST [grant no. JPMJPF2007]).

## Data Availability

The data that support the findings of this study are available from the corresponding author upon reasonable request.

## References

[1] Ridtitid W , Coté GA , Leung W , et al. Prevalence and risk factors for musculoskeletal injuries related to endoscopy. Gastrointest Endosc. 2015;81:294‐302.e4. doi:10.1016/j.gie.2014.06.036 25115360

[2] Matsuzaki I , Ebara T , Tsunemi M , et al. Effects of endoscopy‐related procedure time on musculoskeletal disorders in Japanese endoscopists: A cross‐sectional study. Endosc Int Open. 2021;9:E674‐E683. doi:10.1055/a-1352-3850 33937507 PMC8062226

[3] Morais R , Vilas‐Boas F , Pereira P , et al. Prevalence, risk factors and global impact of musculoskeletal injuries among endoscopists: A nationwide European study. Endosc Int Open. 2020;8:E470‐E480. doi:10.1055/a-1038-4343 32258368 PMC7089795

[4] Shergill AK , Rempel D , Barr A , et al. Biomechanical risk factors associated with distal upper extremity musculoskeletal disorders in endoscopists performing colonoscopy. Gastrointest Endosc. 2021;93:704‐711.e3. doi:10.1016/j.gie.2020.11.001 33160978

[5] Niu J , Wang X , Wang D , Ran L . A novel method of human joint prediction in an occlusion scene by using low‐cost motion capture technique. Sensors (Basel). 2020;20:1119. doi:10.3390/s20041119 PMC7070687 32085653

[6] Seo NJ , Fathi MF , Hur P , Crocher V . Modifying Kinect placement to improve upper limb joint angle measurement accuracy. J Hand Ther. 2016;29:465‐473. doi:10.1016/j.jht.2016.06.010 27769844 PMC6701865

[7] Cai L , Liu D , Ma Y . Placement recommendations for single Kinect‐based motion capture system in unilateral dynamic motion analysis. Healthcare. 2021;9:1076. doi:10.3390/healthcare9081076 34442213 PMC8392214

[8] Galna B , Barry G , Jackson D , Mhiripiri D , Olivier P , Rochester L . Accuracy of the Microsoft Kinect sensor for measuring movement in people with Parkinson's disease. Gait Posture. 2013;39:1062‐1068. 10.1016/j.gaitpost.2014.01.008 24560691

[9] Dutta T . Evaluation of the Kinect™ sensor for 3‐D kinematic measurement in the workplace. Appl Ergon. 2012;43:645‐649. doi:10.1016/j.apergo.2011.09.011 22018839

[10] Bostelman R , Hong T , Legowik S , Shah M . Dynamic metrology and ASTM E57.02 dynamic measurement standard. J CMSC. 2017;12:314‐315. 30997363 PMC6463317

[11] Nakano N , Sakura T , Ueda K , et al. Evaluation of 3D Markerless motion capture accuracy using OpenPose with multiple video cameras. Front Sports Act Living. 2020;27:50. doi:10.3389/fspor.2020.00050 PMC7739760 33345042

